# Supplementary material for: People, primates and predators in the Pontal: from endangered species conservation to forest and landscape restoration in Brazil's Atlantic Forest
Source: R Soc Open Sci. 2020 Dec 9;7(12):200939. doi: 10.1098/rsos.200939 (PMC7813246; doi:10.1098/rsos.200939)

Supplemental Information

Figure S1. Google Earth images of the changing Pontal do Parapanema landscape from 1984 to 2016.


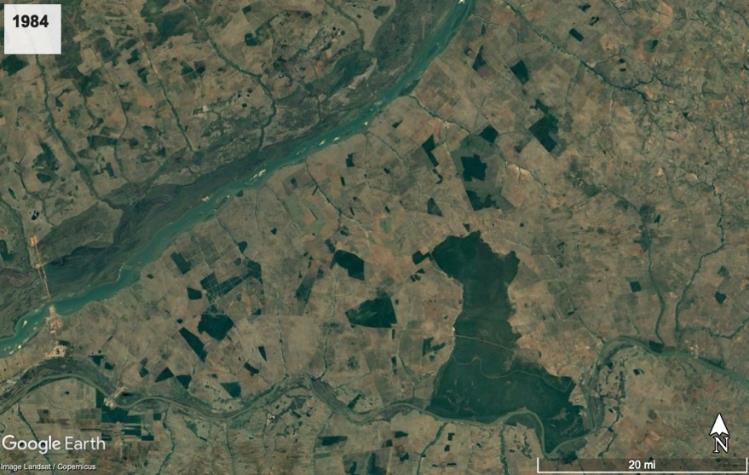

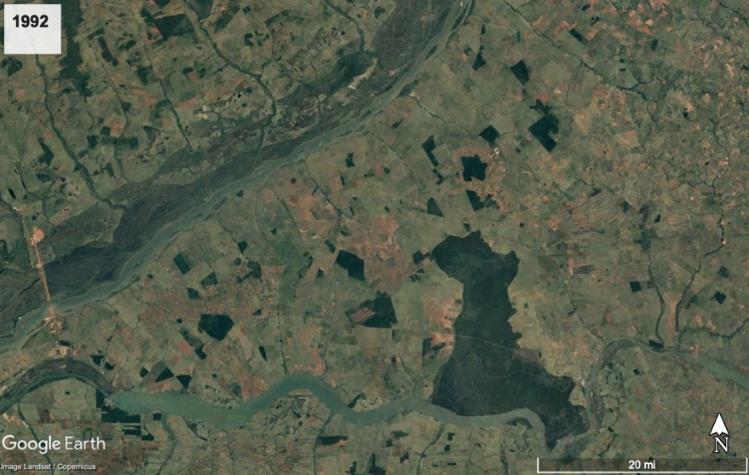

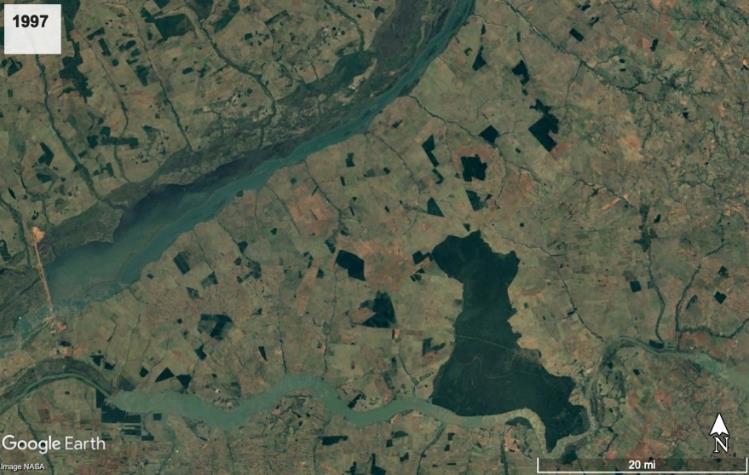

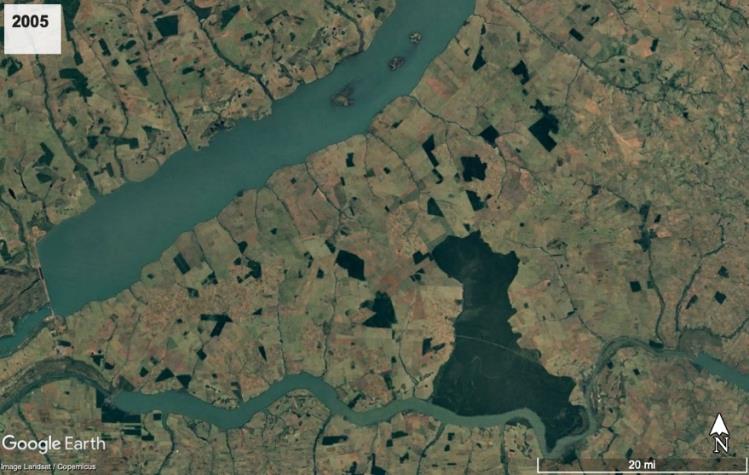

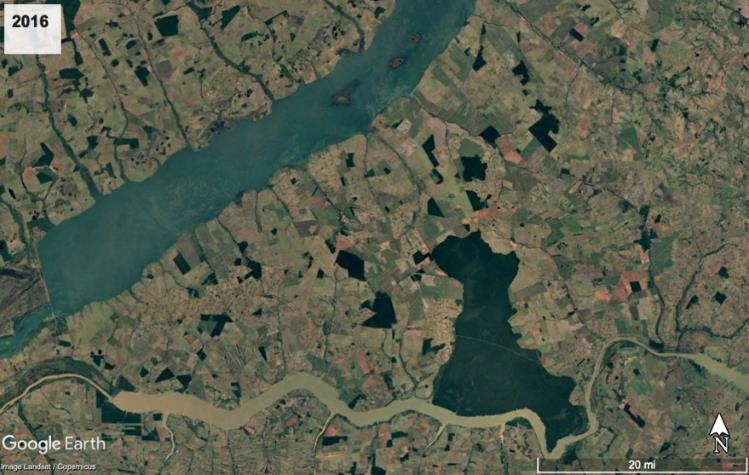


Figure S2. IPÊ’s model of conservation


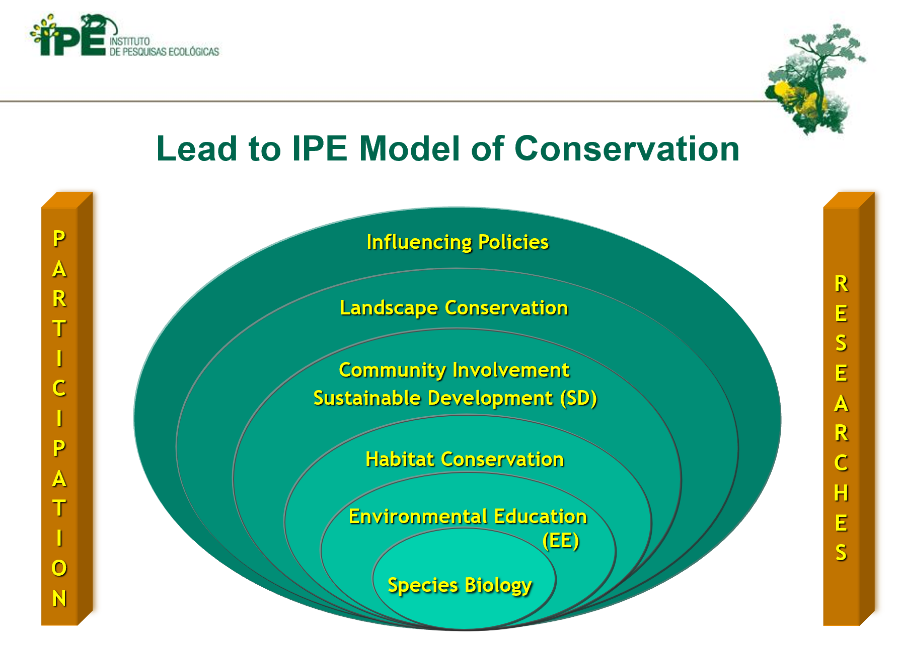

Supplement: Figures S1 and S2 [file rsos200939supp1.docx]
